# To the Medical Profession of Illinois

**Published:** 1865-04

**Authors:** 


### TO THE MEDICAL PROFESSION OF ILLINOIS.

The undersigned, Chairman of the Committee on Practical Medicine of the Illinois State Medical Society, solicits replies to the following series of questions. He would be gratified at receiving papers at large, but, where this is not convenient, succinct answers to the interrogatories here proposed would materially facilitate the purposes of the Committee and the Society. Full credit will in each case be given to respondents. Address, prior to April 20th, Dr. J. ADAMS ALLEN,

Box 4458, Chicago.

1. What is the general character of the surface of the country in your section, (rolling, level, marshy, etc.) ?
2. What is the prevailing direction of the winds ?
3. What is the character of the water used as beverage ?
4. Are the streams sluggish or rapid ?
5. Is the soil loam, clay, gravel, or sand ?
6. Woodland or prairie ?
7. To what extent cultivated ?
8. General occupation of inhabitants ?
9. What particular circumstances exist, which may have an influence on public health ?
10. What are the endemic diseases ?
11. What epidemic forms, the last year ?
12. In what months did they occur ?
13. What ages, sexes, or occupations most affected ?
14. General, or confined to a particular locality ?
15. Proportionate mortality ?
16. Were there any peculiar symptoms, or an absence of usually observed symptoms ?
17. What variation in the relative intensity of the common symptoms was noticed ?
18. What peculiarities observed *post mortem ?*
19. Please state the character of the season during the epidemic, or immediately preceding, and any other circumstance or fact which might explain its prevalence?

20. Any peculiarities of treatment confirmatory or diverse from that laid down in the text books?

